# Role of alpha-lipoic acid in counteracting paclitaxel- and doxorubicin-induced toxicities: a randomized controlled trial in breast cancer patients

**DOI:** 10.1007/s00520-022-07124-0

**Published:** 2022-05-21

**Authors:** Rehab H. Werida, Reham A. Elshafiey, Asser Ghoneim, Sherif Elzawawy, Tarek M. Mostafa

**Affiliations:** 1 Clinical Pharmacy & Pharmacy Practice Department, Faculty of Pharmacy, Damanhour University, Damanhour, Egypt; 2 Pharmacology & Toxicology Department, Faculty of Pharmacy, Damanhour University, Damanhour, Egypt; 3 Clinical Oncology Department, Faculty of Medicine, Alexandria University, Alexandria, Egypt; 4 Clinical Pharmacy Department, Faculty of Pharmacy, Tanta University, Tanta, Egypt

**Keywords:** Alpha-lipoic acid, NCI-CTCAE, Ntx-12, BNP, TNF-α, Neurotensin

## Abstract

**Background and objective:**

Paclitaxel and doxorubicin are associated with neurotoxicity and cardiotoxicity respectively. This study aimed at investigating the role of alpha-lipoic acid (ALA) in counteracting paclitaxel-induced neuropathy and doxorubicin-associated cardiotoxicity in women with breast cancer.

**Patients and methods:**

This randomized double-blind placebo-controlled prospective study included 64 patients with breast cancer who were randomized into control group (*n* = 32) which received 4 cycles of doxorubicin plus cyclophosphamide (every 21 days) followed by weekly doses of paclitaxel for 12 weeks plus placebo tablets once daily and ALA group (*n* = 32) which received the same chemotherapeutic regimen plus ALA 600 once daily for 6 months. Patients were assessed by National Cancer Institute Common Terminology Criteria for Adverse Events (NCI-CTCAE version 4.0) for grading of neuropathy and by 12-item neurotoxicity questionnaire (Ntx-12). The assessment included also echocardiography and evaluation of serum levels of brain natriuretic peptide (BNP), tumor necrosis factor-alpha (TNF-α), malondialdehyde (MDA), and neurotensin (NT). Data were analyzed by paired and unpaired *t-*test, Mann–Whitney *U* test, and chi-square test.

**Results:**

As compared to placebo, ALA provoked significant improvement in NCI-CTCAE neuropathy grading and Ntx-12 score after the end of 9^th^ and 12^th^ weeks of paclitaxel intake (*p* = 0.039, *p* = 0.039, *p* = 0.03, *p* = 0.004, respectively). At the end of the chemotherapy cycles, ALA resulted in significant decline in serum levels of BNP, TNF-α, MDA, and neurotensin (*p* < 0.05) as compared to baseline data and placebo.

**Conclusion:**

Alpha-lipoic acid may represent a promising adjuvant therapy to attenuate paclitaxel-associated neuropathy and doxorubicin-induced cardiotoxicity in women with breast cancer.

**Trial registration:**

ClinicalTrials.gov: NCT03908528.

**Supplementary information:**

The online version contains supplementary material available at 10.1007/s00520-022-07124-0.

## Introduction

Breast cancer represents the most frequently diagnosed malignancy and the second most common cause of cancer death worldwide [1]. Paclitaxel and doxorubicin are cytotoxic agents that are commonly used for treatment of breast cancer. Despite their effectiveness, both paclitaxel and doxorubicin are associated with cumulative and potential neurotoxicity and cardiotoxicity respectively [2, 3].

Paclitaxel-induced peripheral neuropathy (PN) is a consequence of activation of the inflammatory cascade with subsequent increased pro-inflammatory cytokine production including tumor necrosis factor-α (TNF-α), interleukin-1β (IL-1β), and interleukin-6 (IL-6) [4]. Moreover, paclitaxel can upregulate matrix metalloproteinase-3 (MMP3) which plays an important role in the inflammatory and degenerative processes following nerve injury [5]. Oxidative stress plays a critical role in doxorubicin-associated cardiotoxicity through direct cellular damage, induction of apoptosis, and activation of nuclear factor-kappa B (NF-ĸB) which in turn stimulates the production and release of inflammatory mediators [6].

Tumor necrosis factor-alpha (TNF-α) is a well-known inflammatory cytokine and potential mediator for the development of peripheral neuropathy [7]. Tumor necrosis factor-alpha (TNF-α) signaling-dependent vascular inflammation is among the candidate targets for mitigating doxorubicin-associated aortic stiffening and cardiotoxicity [8]. Malondialdehyde (MDA) is one of the end products of lipid peroxidation and represents a useful biomarker for oxidative stress which was identified to be one of the main mechanisms involved in neuropathy [9] and chemotherapy-induced cardiotoxicity [10]. Neurotensin is an endogenous neuropeptide involved in modulation of pain signal transmission and perception [11]. Neurotensin stimulates mast cells which in turn release numerous neurosensitizing and pro-inflammatory mediators with subsequent exacerbation of neuropathic pain [12]. Brain natriuretic peptide (BNP) is secreted by the heart in response to ventricular hypertrophy [13]. There is a strong correlation between BNP level and both the degree of decline in left ventricular ejection fraction [14] and the increase in the left ventricular end-diastolic diameters in patients receiving doxorubicin [15].

Alpha-lipoic acid has been proposed to have anti-oxidant and anti-inflammatory activities [16]. Alpha-lipoic acid was reported to exert beneficial effects in many conditions including cardiovascular disease, cardiovascular-related complications [17], and diabetes associated polyneuropathy [18].

Alpha-lipoic acid (ALA) was reported to have antioxidant activity through scavenging reactive oxygen species (ROS), regenerating endogenous antioxidants including glutathione, vitamin E, and C and through its metal chelation activity [19, 20]. Alpha-lipoic acid (ALA) effectively inhibits nuclear factor-kappa B with subsequent decreasing pro-inflammatory cytokines production (TNF-α, IL-6) and increasing the release of anti-inflammatory cytokines such as interleukin-10 [21]. ALA was reported to be safe and effective in the treatment of symptomatic diabetic neuropathy [22]. A former pre-clinical study reported that ALA can protect the heart against doxorubicin-induced cardiotoxicity through attenuation of oxidative stress [23].

In this context, the aforementioned data encouraged us to run the current study which aimed at evaluating the protective role of alpha-lipoic against paclitaxel-induced neurotoxicity and doxorubicin-induced cardiotoxicity in women with stage II and stage III breast cancer. The primary outcome was the percentage of patients with peripheral sensory neuropathy grade > 2 and the variation of 12-item neurotoxicity questionnaire (Ntx-12) total score. The secondary outcome was the changes in both echocardiographic findings and serum levels of tumor necrosis factor-alpha (TNF-α), malondialdehyde (MDA), neurotensin (NT), and brain natriuretic peptide (BNP).

## Patients and methods

This randomized double-blind placebo-controlled prospective study was conducted between March 2019 and October 2020 on 64 women with stage II and stage III breast cancer who were recruited from Tanta Oncology Center and Alexandria Main University Hospital, Clinical Oncology Department. The study was performed in accordance with the ethical standards of Helsinki declaration in 1964 and its later amendments. The study was approved by both Research Ethics Committee of Damanhur University (Approval number: 219PP10) and Research Ethics Committee of Ministry of Health (Approval Number: 13–2019/4). The study was registered as clinical trial on ClinicalTrials.gov with ID: NCT03908528. All participants gave their written informed consent. All enrolled women were middle-eastern and Egyptians which refers to the ethnic group and the nationality. The inclusion criteria were adult patients (age ≥ 18 and < 70 years old) with biopsy-confirmed diagnosis breast cancer and with stage II and stage III breast cancer according to the American Joint Committee on Cancer (TNM staging system of breast cancer) [24, 25]. Patients with performance status (< 2) according to Eastern Cooperative Oncology Group (ECOG) were included in the study. Patients with adequate hematologic parameters (absolute neutrophil count ≥ 1.5 × 10^9^/L, platelet count ≥ 100 × 10^9^/L, hemoglobin level ≥ 10 g/dl), adequate liver function (serum bilirubin < 1.5 mg/dl), and adequate renal function (serum creatinine < 1.5 mg/dl, creatinine clearance (CrCl) > 45 ml/min) were also included in the study. The exclusion criteria were women with prior exposure to anthracyclines and neurotoxic agents (Cis-platin, vincristine, paclitaxel, docetaxel, foscarnet, isonicotinic acid hydrazide “INH,”, etc.) in the last 6 months and women with evidence of metastasis at the initial assessment. The exclusion criteria also included concomitant use of antioxidant vitamins (vitamin A, C, E), anticonvulsants, tricyclic antidepressants, other medications used for neuropathic pain medication (gabapentin, lamotrigine, carbamazepine), presence of clinical evidence for severe cardiac illness (angina pectoris, uncontrolled hypertension, arrhythmias, and left ventricular ejection fraction < 50%), and pre-existing peripheral neuropathy resulting from other causes such as diabetes and brain disorders. Women with diabetes, women with inflammatory diseases (ulcerative colitis, rheumatoid arthritis), pregnant and breast-feeding women, and women who were candidates for monoclonal antibodies such as Trastuzumab (HER2 positive patients) were also excluded from the study.

The patients were randomized through sealed envelopes method into two groups: group 1 (control group; *n* = 32) which received four cycles of AC regimen (each cycle was given every 21 day and included doxorubicin 60 mg/m^2^ which was diluted with 250 ml normal saline and administered intravenously over 30 min and cyclophosphamide 600 mg/m^2^ which was diluted with 500 ml normal saline and administered intravenously over 60 min) followed by 12 cycles of paclitaxel (cycles were given in weekly basis and each cycle included paclitaxel 80 mg/m^2^ which was diluted with 500 ml normal saline and administered by intravenous infusion over 90 min) plus placebo tablets and group 2 (ALA group; *n* = 32) which received the same chemotherapy regimen plus ALA 600 mg once daily for 6 months.

The dose of alpha-lipoic acid used during the current study (600 mg daily) was selected depending up-on some previous studies and demonstrated that an oral dose of 600 mg alpha-lipoic acid daily improved neuropathic symptoms in diabetic patients with polyneuropathy [18] and provided cardio-protective effect with subsequent improvement of subclinical left ventricular dysfunction in asymptomatic patients with type 1 diabetes [26]. Additionally, the implicated dose of alpha-lipoic acid during the current study comes in accordance with a recently published study reported efficacy of alpha-lipoic acid supplementation in chemotherapy-induced peripheral neuropathy [27].

It is worth mentioning that intravenous 5-HT3 antagonist plus pantoprazole were administered to all participants before each cycle as prophylactic therapy against chemotherapy-induced nausea, vomiting, and gastrointestinal upset. All patients were submitted to complete blood count analysis before each chemotherapy cycle. The investigator was provided with a sealed randomization code for each available medication generated by an independent researcher. Blindness was maintained by ensuring that the appearance and the color of placebo and ALA tablets were identical.

### Demography and anthropometric measurements

All participants were submitted to demographic data collection (age), medication history taking, physical examination, and measurement of weight and height with subsequent calculation of body mass index: BMI = [weight (kg) ÷ height^2^ (m)].

### Clinical assessment of paclitaxel induced peripheral sensory neuropathy

Grading of paclitaxel induced peripheral sensory neuropathy was assessed using the National Cancer Institute Common Terminology Criteria for Adverse Events (NCI-CTCAE version 4.0, 2009) [28] which classified the peripheral sensory neuropathy into 5 grades. Grade (1) mild and asymptomatic; grade (2) moderate symptoms; grade (3) severe symptoms; grade (4) life-threatening consequences; and grade (5) death as illustrated in Appendix Table (I). All participants in the two study groups were clinically assessed and graded for peripheral sensory neuropathy before starting of paclitaxel and after the 3^rd^, 6^th^, 9^th^, and 12^th^ week of paclitaxel administration. Additionally, a 12-item neurotoxicity questionnaire (Ntx-12) from the validated functional Assessment of Cancer Therapy/Gynecologic Oncology Group (FACT/GOG-Ntx-12; version 4: Appendix Table II) was used to evaluate the severity and impact of neuropathy on patients’ life. Each item in Ntx-12 questionnaire is represented by a Likert-type scale ranging from 0 to 4 with 0 refers to not at all, 1 means little a bit, 2 means somewhat, 3 means quite a bit, and 4 refers to very much. The score was calculated using the reverse item as follows: (4-item response = item score). The sum of individual items scores is multiplied by 12 and then divided by number of items answered. The higher Ntx-12 score, the better quality of life (QOL).

### Clinical assessment of doxorubicin induced cardiotoxicity

Echocardiographic assessment of chemotherapy-related cardiotoxicity was done at baseline and after the last doxorubicin/cyclophosphamide (AC) cycle. Echocardiographic assessment was done using Vivid 5 ultrasound machine (Horten, Norway).

### Blood sampling and biochemical analyses

Approximately 1 h before starting the first chemotherapy cycle (baseline) and 1 h after the last AC cycle (for assessment of BNP) and the last paclitaxel cycle (for assessment of neurotensin, MDA, and TNF-α), 5 ml of venous blood was withdrawn by antecubital venipuncture from each participant between 8:30 and 10:30 am into a plain test tube and centrifuged at 3000 rpm for 10 min. The separated sera were frozen at − 80 °C until analysis of the biochemical parameters. Serum levels of brain natriuretic peptide (BNP), tumor necrosis factor-alpha (TNF-α), and neurotensin (NT) were determined by double antibody sandwich enzyme-linked immune-sorbent assay (Sun Red, Biological Technology Co., Ltd, Shanghai, China). Malondialdehyde (MDA) as oxidative stress marker was determined through the method formerly described by Draper and Hadly [29].

### Assessment of participants’ adherence and drugs tolerability

Alpha-lipoic acid and placebo tablets were provided on monthly intervals and the participants’ adherence was assessed through the medications refilling rate and through pill counts. Participants were also followed up by telephone calls and through direct meetings during chemotherapy cycles to assess their adherence and report any drug-related adverse effects using adverse effect questionnaire. The adverse effects were also collected from the patients’ laboratory data and patient sheet. The patients were asked about any adverse effects related to all study medications. Because the two groups were similar in chemotherapy regimen and were different only in ALA and placebo, so we can attribute any difference in the reported side effects to ALA particularly headache, nausea, abdominal discomfort, or abdominal pain. Patients were considered non-adherent and excluded from the study if consumed less than 90% of the study medication at any month of the study duration.

### Sample size calculation

The required sample size was calculated using G*Power software version 3.1.0 (Institut für Experimentelle Psychologie, Heinrich Heine Universität, Dusseldorf, Germany). It was estimated that a total sample size of 64 patients would have a power of 95.2% to detect a medium to large effect size of 0.92 in the outcome measured.

### Statistical analysis

Statistical analysis was done by the statistical software package SPSS version 25 (SPSS Inc., Chicago, IL, USA). Data were tested for normality using Kolmogorov–Smirnov and Shapiro–Wilk tests. Normally distributed data were compared using Student *t*-test. Non-normally distributed data were compared using Mann–Whitney *U* test. Also, Mann–Whitney *U* or Chi-Square test was used to compare non-parametric data between the two arms including the neuropathy grading and the Ntx-12 total scores as appropriate. Chi-square test was used for categorical data. The significance level was set at *p* < 0.05.

## Results

**Fig. 1:**
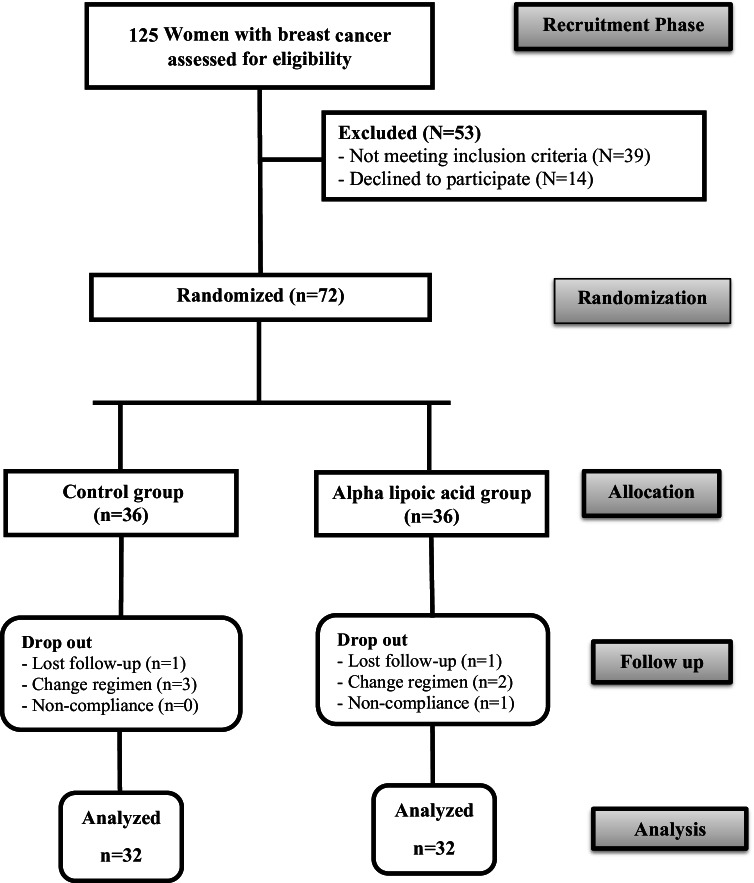
Flow chart of patients enrollment, randomization, and follow-up during the study

Patients’ enrollment, randomization, and follow-up during the course of the study are demonstrated in Fig. (1). A total number of 125 patients with stage II and stage III breast cancer were assessed for eligibility and 53 women were excluded (not meeting the inclusion criteria (*n* = 39) and declined to participate (*n* = 14)). Therefore, 72 patients were randomized into the two study groups (*n* = 36 for each group). During the follow-up period, a total number of 8 women were dropout in both groups secondary to loss of follow-up (*n* = 2), change of chemotherapy regimen (*n* = 5), and non-compliance (*n* = 1). The final analysis included 64 patients with 32 women in each group.

At baseline, the two study groups were statistically similar in respect to age, anthropometric data (weight, height, and body mass index), menopausal status, stages of disease, types of surgery, ECOG score, fasting blood glucose level, ejection fraction (EF), brain natriuretic peptide (BNP) level, neuropathy grade (grade 0), Ntx-12 total score, and cumulative doses of chemotherapeutic agents as illustrated in Table (1). In addition, there was non-significant difference in biological markers (TNF-α, MDA, and neurotensin) between the two study groups as demonstrated in Table (2).

**Table 1:** Baseline demographic data and clinical characteristics of the two study groups

Demographic data	Control group (*n* = 32)	ALA group (*n* = 32)	*p*-value
**Age (years) **	49.56 ± 9.18 (36–63)	48.19 ± 7.83 (36–62)	0.521
**Weight (kg)**	80.50 ± 12.56	79.16 ± 13.31	0.679
**Height (cm)**	160.91 ± 6.31	160.06 ± 6.89	0.611
**BMI (kg/m**^**2**^**)**	31.23 ± 5.48	31.01 ± 5.45	0.873
**BSA (m**^**2**^**)**	1.89 ± 0.16	1.87 ± 0.17	0.604
**Menopausal status**			
Premenopausal	18 (56.3%)	20 (62.5%)	0.611
Postmenopausal	14 (43.8%)	12 (37.5%)	
**Stage of disease**			
II	17 (53.1%)	16 (50%)	0.802
III	15 (46.9%)	16 (50%)	
**Type of surgery**			
MRM	18 (56.3%)	20 (62.5%)	0.611
BCS	14 (43.8%)	12 (37.5%)	
**ECOG score**			
0	11 (34.4%)	9 (28.1%)	0.590
1	21 (67.6%)	23 (71.9%)	
**FBG (mg/dl)**	89.53 ± 8.08	88.03 ± 9.33	0.494
**Ejection fraction (%)**	67 (60–73)	65.5 (58–71)	0.081
**BNP (ng/L)**	57.04 ± 18.66	56.66 ± 23.11	0.943
**Neuropathy grade**	0	0	1
**Ntx-12 score**	45.06 ± 1.50	45.16 ± 1.57	0.838
**Mean cumulative doses of chemotherapeutic agents**			
Doxorubicin	453.77 ± 37.60	448.69 ± 40.40	0.604
Cyclophosphamide	4537.73 ± 375.89	4486.87 ± 403.99	0.604
Paclitaxel	1815.09 ± 150.36	1794.75 ± 161.60	0.604

Data are presented as mean ± standard deviation, range, number, and percentage

Data were analyzed by unpaired *t* test, Mann–Whitney *U* test, or chi-square test as appropriate

Significance level was set at *p* < 0.05

*BMI*, body mass index; *BSA*, body surface area; *MRM*, modified radical mastectomy; *BCS*, breast-conserving surgery; *ECOG*, Eastern Cooperative Oncology Group; *FBG*, fasting blood glucose; *BNP*, brain natriuretic peptide; *Ntx-12 score*, neutotoxicity-12 questionnaire total score

**Table 2:** Ejection fraction and biological markers of the two study groups before and after chemotherapy cycles

Parameters	Control group (*n* = 32)	ALA group (*n* = 32)	*p* value
**EF (%)**			
Before chemotherapy	67 (60–73)	65.5 (58–71)	0.081
After chemotherapy	61.5 (55–68)	58.5 (55–69)	0.113
Paired *t* test	< 0.0001^*^	< 0.0001^*^	
**BNP (ng/L)**			
Before chemotherapy	57.04 ± 18.66	56.66 ± 23.11	0.943
After chemotherapy	88.48 ± 35.64	48.56 ± 17.71	< 0.0001^*^
Paired *t* test	< 0.0001^*^	0.039^*^	
**TNF-α (pg/L)**			
Before chemotherapy	67.51 ± 19.46	74.18 ± 27.02	0.261
After chemotherapy	120.43 ± 36.24	79.07 ± 36.88	< 0.0001^*^
Paired *t* test	< 0.0001^*^	0.562	
**MDA (nmol/ml)**			
Before chemotherapy	7.16 ± 1.80	7.03 ± 2.02	0.788
After chemotherapy	8.36 ± 1.60	6.12 ± 1.81	0.0001^*^
Paired *t* test	0.001^*^	0.012^*^	
**NT (Pmol/L)**			
Before chemotherapy	68.27 ± 16.20	70.19 ± 19.89	0.673
After chemotherapy	88.57 ± 19.50	60.11 ± 11.55	< 0.0001^*^
Paired *t* test	< 0.0001^*^	0.002^*^	

Data are presented as mean ± standard deviation, median (range)

Data were analyzed by *t* test, Mann–Whitney *U* test, or Wilcoxon-signed ranks test as appropriate

Significance level was set at *p* < 0.05

*EF* ejection fraction, *BNP* brain natriuretic peptide, *TNF-α* tumor necrosis factor-alpha, *MDA* malondialdehyde, *NT* neurotensin

^*^Significant

**Fig. 2:**
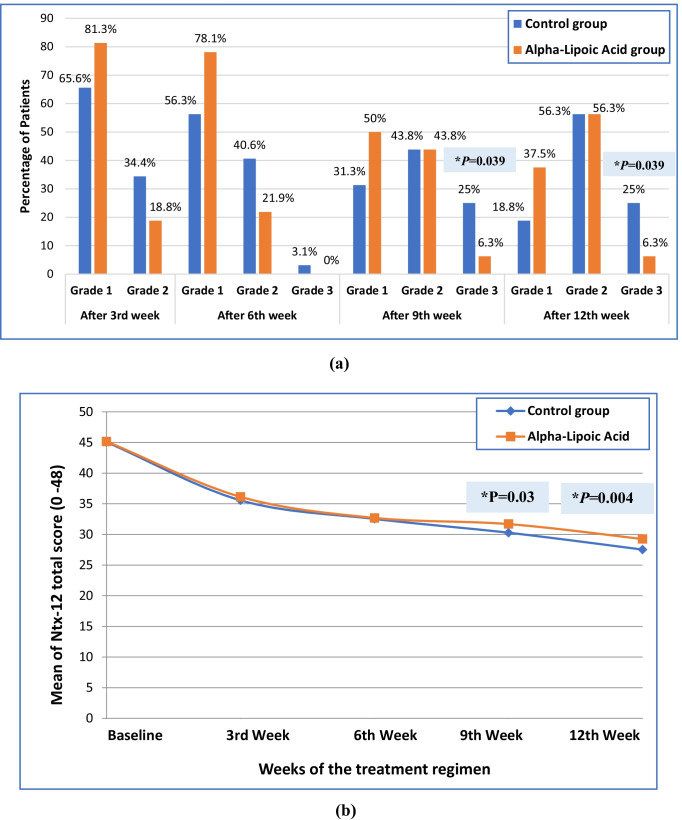
Clinical assessment of paclitaxel induced peripheral sensory neuropathy. Data were analyzed by Mann–Whitney *U* test or chi-square test as appropriate. Significance level was set at *p* < 0.05. (**a**) Neuropathy grading for the two study groups throughout the course of treatment. (**b**) Ntx-12 total score for the two study groups throughout the course of treatment

During the course of paclitaxel treatment, there was an increase in neuropathy grades and a decline in FACT/GOG-Ntx-12 questionnaire total score in both study groups. There was non-significant difference in neuropathy grades and FACT/GOG-Ntx-12 questionnaire total score between the two study groups during the course of paclitaxel cycles except after the 9^th^ and 12^th^ week. After the 9^th^ and 12^th^ week, the percentage of patients with grade 3 peripheral sensory neuropathy was significantly lower in alpha-lipoic acid group as compared to the control group (6.3 versus 25%; *p* = 0.039; and 6.3 versus 25%; *p* = 0.039, respectively). However after the 9^th^ and 12^th^ week, there was non-significant variation between the two study groups in the percentage of patients with grade 1 peripheral sensory neuropathy (31.3 versus 50%; *p* = 0.127; and 18.8 versus 37.5%; *p* = 0.095, respectively) and grade 2 peripheral sensory neuropathy (43.8 versus 43.8%; *p* = 1.000; and 56.3 versus 56.3%; *p* = 1.000, respectively) as shown in Fig. (2a). The FACT/GOG-Ntx-12 questionnaire total score was significantly higher in ALA group as compared to the control group (31.69 ± 2.83 versus 30.28 ± 2.29; *p* = 0.03; and 29.25 ± 2.44 versus 27.53 ± 1.70; *p* = 0.004, respectively). The data obtained Ntx-12 questionnaire total score are presented in Fig. (2b).

Concerning echocardiography findings and as compared to the baseline data, both groups showed significant decline in ejection fraction (EF) after intervention (*p* < 0.0001 and *p* < 0.0001, respectively). Furthermore, the ejection fraction showed non-significant variation between the two study groups after treatment (*p* = 0.113) as illustrated in Table (2).

Regarding the data obtained with the biological markers and as compared to baseline data, the control group showed significant elevation in BNP, TNF-α, MDA, and neurotensin serum levels after intervention (*p* < 0.0001, *p* < 0.0001, *p* = 0.001, and *p* < 0.0001, respectively). On the other hand, after intervention, ALA group showed significant decline in BNP, MDA, and neurotensin serum levels (*p* = 0.039, *p* = 0.012, and *p* = 0.002, respectively) with non-significant elevation in TNF-α serum level (*p* = 0.56). The comparison between the two study groups after intervention revealed that ALA group showed significant decline in BNP, TNF-α, MDA, and neurotensin serum levels as compared to the control group (*p* < 0.0001, *p* < 0.0001, *p* = 0.0001, and *p* < 0.0001, respectively). The data obtained with the biological markers are shown in Table (2).

Regarding the reported adverse effects, there was non-significant difference between the two study groups (*p* > 0.05). These result means that the addition of ALA to chemotherapy was not associated with increased incidence of adverse effects. In this context, alpha-lipoic acid supplement 600 mg daily was well tolerated. The reported adverse effects are demonstrated in Table (3).

**Table 3:** Reported adverse events within the two study groups

	**Group II (placebo)** **(*n* = 32)**		**Group I (ALA)** **(*n* = 32)**		***p* value**
	**No**	**%**	**No**	**%**	
Nausea	2	6.3	2	6.3	1.00
Abdominal pain	1	3.1	2	6.3	0.554
Dizziness	3	9.4	2	6.3	0.641
Headache	2	6.3	3	9.4	0.641
Grade 1 & 2 neutropenia	6	18.8	7	21.9	0.756
Grade 1 & 2 leukopenia	11	34.4	9	28.1	0.590
Infection	5	15.6	3	9.4	0.450
Grade 1 fatigue	27	84.4	24	75.1	0.351
Grade 2 and 3 fatigue	2	6.3	0	0	0.151

Data are presented as number and percent

Data were analyzed by chi-square test

Significance level was set at *p* < 0.05

Grade 1 neutropenia (neutrophils count: 2000–1500/mm^3^); grade 2 neutropenia (neutrophils count: 1000– < 1500/mm^3^); grade 1 leukopenia (WBCS count: < 4000–3000/mm^3^); grade 2 leukopenia (WBCS count: < 3000–2000/mm^3^); grade 1 fatigue (fatigue relieved by rest); grade 2 fatigue (fatigue not relieved by rest and limits instrumental activity); grade 3 fatigue (fatigue not relieved by rest and limits self-care activity)

*GIT* gastrointestinal tract

## Discussion

This study aimed at evaluating the protective role of ALA against paclitaxel-induced peripheral sensory neuropathy and doxorubicin-associated cardiotoxicity in patients with stage II and stage III breast cancer.

By the end of the 9^th^ and 12^th^ weeks of paclitaxel administration, the percentage of patients with grade 3 peripheral sensory neuropathy was significantly lower in ALA group than those in the control group and the FACT/GOG-Ntx-12 total score was significantly higher in ALA group as compared to the control group. The favorable effect of ALA on Ntx-12 total score was clinically translated by improvement in severity of neuropathy and its impact on patients’ life including improvement in joint pain, trouble associated with handling button and walking, and discomfort of hands and feet. Our findings obtained with grading of neuropathy and Ntx-12 total score indicate the protective effect of ALA against paclitaxel-induced peripheral sensory neuropathy which may be attributed to its anti-inflammatory and antioxidant effects. It has been reported that paclitaxel-induced peripheral neuropathy is mainly related to activation of inflammatory cascade [4]. Additionally, ALA as antioxidant was reported to exert neuro-protective effect and can be used to treat taxanes-induced cumulative polyneuropathy [30]. Furthermore, the neuro-protective effect of ALA may be attributed to its favorable effect on neurotensin level which can stimulate mast cells with subsequent secretion of neuro-sensitizing and pro-inflammatory mediators which exacerbate neuropathic pain [31]. Our findings are compatible with the findings of a former study postulated that co-administration of ALA with oxaliplatin in patients with colorectal cancer resulted in prevention of oxaliplatin-related neuropathy in 53% of patients [32]. Our findings are also comparable to the findings of a previous study and revealed that ALA reduced docetaxel / cisplatin-induced peripheral neuropathy in patients with gastric cancer [30]. Furthermore, our results come in parallel with a former study that reported presence of statistically significant difference in FACT/GOG-Ntx-12 total score between ALA group and the control groups in patients received chemotherapy-induced peripheral neuropathy [33]. In the contrary, our result seems inconsistent with a previously reported study conducted on patients received platinum-based chemotherapy and revealed absence of statistically significant difference between ALA group and placebo group regarding their effects on peripheral neuropathy [34].

Although ALA exerts beneficial effect on BNP level, there was non-significant difference in ejection fraction between the control group and ALA group after intervention. Our former finding may be attributed to the notion that left ventricular (LV) ejection fraction is not a sensitive tool for early detection of sub-clinical cardiac diseases [35]. In this context, the circulating cardiac biomarkers represent reproducible and sensitive tool for early detection of chemotherapy-induced cardiac toxicity [36]. Our former result concerning ejection fraction seems in agreement with a previously reported study and demonstrated that there was no significant difference in ejection fraction between ALA group and the control group in patients with previous experience of transient takotsubo cardiomyopathy [37].

Conflicting data were reported about the effects of alpha-lipoic acid on brain natriuretic peptide (BNP), tumor necrosis factor-alpha (TNF-α), malondialdehyde (MDA), and neurotensin. It has been demonstrated that there was an elevation in BNPlevel during treatment with doxorubicin [38] and ALA can ameliorate doxorubicin-associated cardiotoxicity [22] and can reduce cardiovascular events in patients on hemodialysis [39]. In contrast, a previous study revealed that there was no significant difference in BNP level between ALA group and the control group 12 months after treatment in patients with a previous experience of transient takotsubo cardiomyopathy [37]. Regarding TNF-α, some previous studies revealed that ALA decreased TNF-α level in type-1 diabetic patients with subclinical left ventricular dysfunction [26] and in patients with atrial fibrillation [40]. However, other authors reported lack of beneficial effect of ALA on serum TNF-α level in patients with rheumatoid arthritis [41]. A previous clinical study reported that ALA 300 mg twice daily for 4 months significantly decreased MDA in type-1 diabetic patients with subclinical left ventricular dysfunction [26]. In the contrary, ALA supplementation did not produce any significant change in malondialdehyde serum level in patients on hemodialysis [42].

During the current study and as compared to baseline data, the control group showed significant elevation in BNP, TNF-α, malondialdehyde (MDA), and neurotensin serum levels after intervention. These results can be attributed to doxorubicin-associated cardiotoxicity [3], doxorubicin, and paclitaxel associated upregulation of pro-inflammatory cytokines including TNF-α [43, 44], doxorubicin-induced oxidative stress [23, 45], and paclitaxel-induced peripheral neuropathy secondary to activation of inflammatory cascade [4, 11]. Concerning ALA group, supplementation with ALA showed a significant decline in BNP serum level as compared to its baseline and to the control group. The beneficial effect of ALA on BNP level can be explained on the basis of its antioxidant and anti-inflammatory activities [19, 46] which in turn may provide cardio-protective effect. Furthermore, ALA can decrease serum BNP and cardiac hypertrophy through attenuating the mRNA and protein levels of C/EBPβ which is responsible for cardiomyocyte hypertrophy [47]. After intervention, ALA group showed a significantly lower TNF-α level as compared to the control group which may be attributed to the ability of ALA to suppress nuclear factor-kappa B with subsequent decreased production of pro-inflammatory cytokines including TNF-α [21]. In addition, post-treatment, ALA group showed significantly lower MDA serum level as compared to its baseline data and to the control group. The favorable effect of ALA on MDA level is attributed to its antioxidant activity with subsequent scavenging reactive oxygen species, regenerating endogenous antioxidants including glutathione, vitamin E, vitamin C, and its metal chelation activity [19, 20]. Furthermore, ALA group showed a significantly lower neurotensin level as compared to its baseline data and to the control group. This beneficial effect of ALA on neurotensin may be related to the notion that ALA may effectively decrease the production of pro-inflammatory cytokines including TNF-α, IL-1β, and IL-6 [21] which provoke inflammatory pain stimuli with subsequent enhancement of neurotensin release [4, 11]. The overall favorable effect of ALA on BNP, TNF-α, and MDA comes in accordance with some previous studies [26, 39, 40] and in contradiction with others [37, 41, 42]. These contradictory results may be attributed to the difference in the underlying disease state, difference in the implicated doses of ALA, and the variation in the treatment duration.

The overall data obtained with the current study revealed that ALA was well tolerated and effective as adjuvant therapy against chemotherapy-induced toxicity in patients with breast cancer. The current study did not aim at evaluation of the effect of ALA on the efficacy of chemotherapeutic agents secondary to its design and the need for long follow-up period to assess the interaction between chemotherapy and ALA. In this context, we recommend further longitudinal studies that aim at examining the effect of ALA on the efficacy of chemotherapeutic agents since conflicting data exist about the interaction between ALA and some types of chemotherapy. Some authors hypothesized that antioxidant therapies may antagonize antitumor effects by reducing oxidative damage [48]. On the other hand, several case reports have been published suggesting that ALA typically in combination with other agents may have anticancer activity [49, 50].

The points of strength of the current study include its design as a placebo controlled double-blind clinical trial. In addition and to the best of authors’ knowledge, this clinical study may be the first one amid at evaluating the effect of alpha-lipoic acid on paclitaxel and doxorubicin-induced neurotoxicity and cardiac toxicity and its effect on serum neurotensin level in women with stage II and stage III breast cancer. However, the current study has some limitations including the relatively small sample size and the relatively short follow-up period. The sample size used during the current study may not be large enough to detect an effect on cardiotoxicity, due to the low frequency of this toxicity at the implicated doses of doxorubicin. Furthermore, the use of fixed dose for alpha-lipoic acid and the implications of per protocol analysis represent other limitations of the study. Therefore, more large scale and more longitudinal studies with implication of different doses of alpha-lipoic acid are still necessary.

## Conclusion

This randomized double-blind placebo controlled prospective clinical study aimed at evaluating the protective role of alpha-lipoic acid against paclitaxel-induced peripheral sensory neuropathy and doxorubicin-related cardiotoxicity in patients with stage II and stage III breast cancer. The results obtained with the current study revealed that the implication of alpha-lipoic acid 600 mg daily as adjunct therapy to doxorubicin and paclitaxel was associated with significant improvement in NCI-CTCAE peripheral sensory neuropathy grading and FACT/GOG-Ntx-12 total score. Additionally, alpha-lipoic acid produced significant decline in the serum levels of brain natriuretic peptide (BNP), inflammatory marker (TNF-α), oxidative stress marker (MDA), and neurotensin (NT). However, alpha-lipoic acid did not produce significant effect on left ventricular ejection fraction as compared to the control group which may be attributed to the small sample size and the low sensitivity of left ventricular ejection fraction as a detection tool for sub-clinical cardiac diseases. In this context, alpha-lipoic acid may provide supportive care in cancer and may represent a promising adjuvant therapy to attenuate paclitaxel-induced peripheral neuropathy and to counteract doxorubicin-induced cardiotoxicity. However, these results warrant further investigations.

## Supplementary Information

Below is the link to the electronic supplementary material.

Supplementary file1 (DOCX 82 KB)

Supplementary file2 (PDF 363 KB)

## Data Availability

The data that support the findings of this study are available upon reasonable request.
