# Supplementary material for: Role of alpha-lipoic acid in counteracting paclitaxel- and doxorubicin-induced toxicities: a randomized controlled trial in breast cancer patients
Source: Support Care Cancer. 2022 May 21;30(9):7281–92. doi: 10.1007/s00520-022-07124-0 (PMC9385783; doi:10.1007/s00520-022-07124-0)

**Appendix Table (I): Grading of neuropathy according to NCT-CTCAE (Version 4; 2009)**

| Adverse Event | Peripheral Sensory Neuropathy |
| --- | --- |
| Grade 1 | Asymptomatic; loss of deep tendon reflexes or paresthesias |
| Grade 2 | Moderate symptoms; limiting instrumental activates of daily living as shopping, handling money, handling phone, |
| Grade 3 | Severe symptoms; limiting self-care activates of daily living as dressing, undressing, bathing, cooking, etc |
| Grade 4 | Life-threatening consequences; urgent intervention indicated |
| Grade 5 | Death |

**Appendix Table (II): Ntx-12 item questionnaire from the validated Functional Assessment of Cancer Therapy/Gynecologic Oncology Group (FACT/GOG;Version 4)**


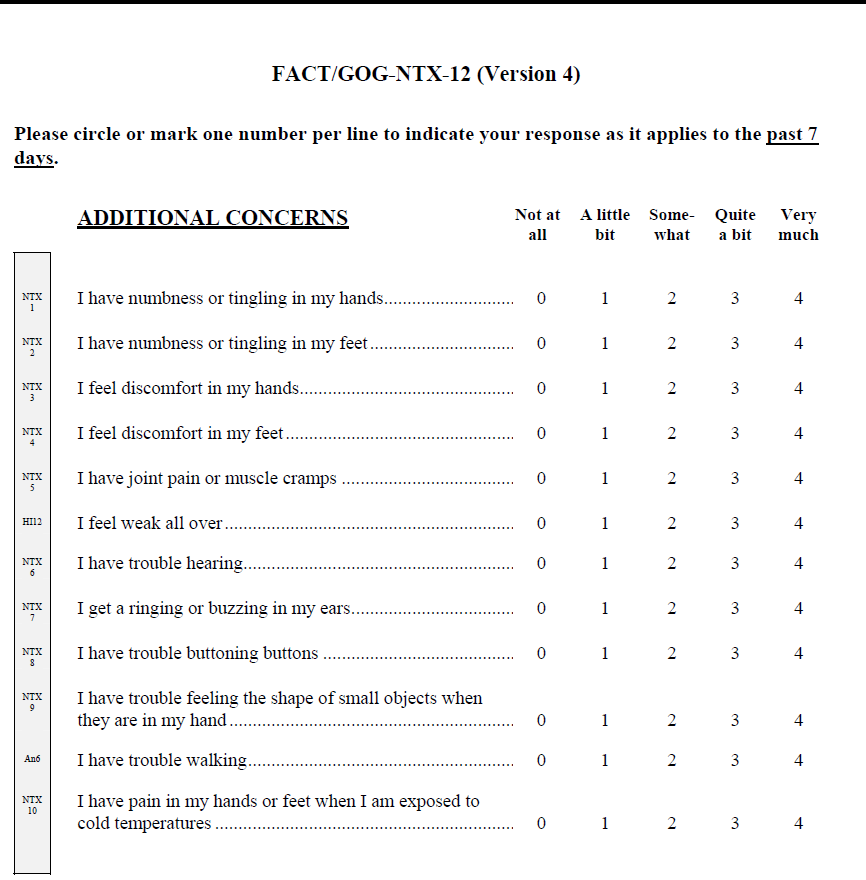

Supplement: Supplementary file 1 — Supplementary file1 (DOCX 82 KB) [file 520_2022_7124_MOESM1_ESM.docx]
