# Supplementary material for: Role of alpha-lipoic acid in counteracting paclitaxel- and doxorubicin-induced toxicities: a randomized controlled trial in breast cancer patients
Source: Support Care Cancer. 2022 May 21;30(9):7281–92. doi: 10.1007/s00520-022-07124-0 (PMC9385783; doi:10.1007/s00520-022-07124-0)
Supplement: Supplementary file 2 — Supplementary file2 (PDF 363 KB) [file 520_2022_7124_MOESM2_ESM.pdf]

| groups | Age (Year) | WT (Kg) | HT (cm) | BSA | BMI | FBG | ECOG | Doxorubicin |
|---|---|---|---|---|---|---|---|---|
| 1.00 | 51.00 | 57.00 | 157.00 | 1.58 | 23.12 | 82.00 | 0.00 | 378.40 |
| 1.00 | 41.00 | 89.00 | 154.00 | 1.95 | 37.53 | 95.00 | 0.00 | 468.29 |
| 1.00 | 39.00 | 94.00 | 157.00 | 2.02 | 38.14 | 100.00 | 1.00 | 485.93 |
| 1.00 | 40.00 | 63.00 | 159.00 | 1.67 | 24.92 | 78.00 | 1.00 | 400.34 |
| 1.00 | 38.00 | 74.00 | 164.00 | 1.84 | 27.51 | 85.00 | 1.00 | 440.65 |
| 1.00 | 61.00 | 66.00 | 165.00 | 1.74 | 24.24 | 90.00 | 0.00 | 417.42 |
| 1.00 | 58.00 | 76.00 | 158.00 | 1.83 | 30.44 | 105.00 | 0.00 | 438.32 |
| 1.00 | 39.00 | 84.00 | 157.00 | 1.91 | 34.08 | 84.00 | 1.00 | 459.36 |
| 1.00 | 59.00 | 81.00 | 153.00 | 1.86 | 34.60 | 80.00 | 1.00 | 445.30 |
| 1.00 | 62.00 | 90.00 | 155.00 | 1.97 | 37.46 | 92.00 | 1.00 | 472.44 |
| 1.00 | 57.00 | 74.00 | 160.00 | 1.81 | 28.91 | 88.00 | 1.00 | 435.25 |
| 1.00 | 49.00 | 92.00 | 175.00 | 2.11 | 30.04 | 78.00 | 0.00 | 507.54 |
| 1.00 | 39.00 | 83.00 | 149.00 | 1.85 | 37.39 | 80.00 | 1.00 | 444.83 |
| 1.00 | 38.00 | 85.00 | 155.00 | 1.91 | 35.38 | 95.00 | 0.00 | 459.13 |
| 1.00 | 54.00 | 100.00 | 172.00 | 2.19 | 33.80 | 90.00 | 0.00 | 524.60 |
| 1.00 | 53.00 | 55.00 | 159.00 | 1.56 | 21.76 | 98.00 | 1.00 | 374.06 |
| 1.00 | 63.00 | 68.00 | 162.00 | 1.75 | 25.91 | 86.00 | 1.00 | 419.83 |
| 1.00 | 55.00 | 91.00 | 168.00 | 2.06 | 32.24 | 95.00 | 1.00 | 494.58 |
| 1.00 | 58.00 | 104.00 | 158.00 | 2.14 | 41.66 | 90.00 | 0.00 | 512.75 |
| 1.00 | 61.00 | 96.00 | 154.00 | 2.03 | 40.48 | 78.00 | 1.00 | 486.36 |
| 1.00 | 49.00 | 85.00 | 158.00 | 1.93 | 34.05 | 88.00 | 1.00 | 463.55 |
| 1.00 | 39.00 | 64.00 | 160.00 | 1.69 | 25.00 | 80.00 | 1.00 | 404.77 |
| 1.00 | 55.00 | 61.00 | 169.00 | 1.69 | 21.36 | 98.00 | 0.00 | 406.13 |
| 1.00 | 37.00 | 80.00 | 172.00 | 1.96 | 27.04 | 108.00 | 1.00 | 469.21 |
| 1.00 | 56.00 | 71.00 | 161.00 | 1.78 | 27.39 | 86.00 | 1.00 | 427.66 |
| 1.00 | 36.00 | 92.00 | 156.00 | 2.00 | 37.80 | 85.00 | 1.00 | 479.20 |
| 1.00 | 51.00 | 88.00 | 169.00 | 2.03 | 30.81 | 82.00 | 1.00 | 487.80 |
| 1.00 | 58.00 | 83.00 | 161.00 | 1.93 | 32.02 | 94.00 | 1.00 | 462.39 |
| 1.00 | 60.00 | 79.00 | 157.00 | 1.86 | 32.05 | 87.00 | 1.00 | 445.48 |
| 1.00 | 44.00 | 86.00 | 170.00 | 2.02 | 29.76 | 94.00 | 1.00 | 483.65 |
| 1.00 | 37.00 | 75.00 | 161.00 | 1.83 | 28.93 | 102.00 | 0.00 | 439.55 |
| 1.00 | 49.00 | 90.00 | 164.00 | 2.02 | 33.46 | 92.00 | 0.00 | 485.96 |
| 2.00 | 58.00 | 110.00 | 164.00 | 2.24 | 40.90 | 80.00 | 1.00 | 537.25 |
| 2.00 | 47.00 | 92.00 | 155.00 | 1.99 | 38.29 | 100.00 | 0.00 | 477.66 |
| 2.00 | 50.00 | 91.00 | 169.00 | 2.07 | 31.86 | 96.00 | 0.00 | 496.05 |
| 2.00 | 55.00 | 66.00 | 147.00 | 1.64 | 30.54 | 80.00 | 1.00 | 393.99 |
| 2.00 | 45.00 | 71.00 | 150.00 | 1.72 | 31.56 | 84.00 | 1.00 | 412.80 |
| 2.00 | 38.00 | 75.00 | 160.00 | 1.83 | 29.30 | 80.00 | 1.00 | 438.18 |
| 2.00 | 41.00 | 79.00 | 164.00 | 1.90 | 29.37 | 98.00 | 0.00 | 455.30 |
| 2.00 | 47.00 | 66.00 | 158.00 | 1.70 | 26.44 | 78.00 | 1.00 | 408.47 |
| 2.00 | 52.00 | 54.00 | 164.00 | 1.57 | 20.08 | 76.00 | 0.00 | 376.43 |
| 2.00 | 49.00 | 102.00 | 170.00 | 2.19 | 35.29 | 74.00 | 0.00 | 526.73 |
| 2.00 | 51.00 | 94.00 | 154.00 | 2.01 | 39.64 | 101.00 | 1.00 | 481.26 |
| 2.00 | 37.00 | 83.00 | 151.00 | 1.87 | 36.40 | 87.00 | 1.00 | 447.80 |
| 2.00 | 36.00 | 74.00 | 159.00 | 1.81 | 29.27 | 95.00 | 1.00 | 433.88 |
| 2.00 | 54.00 | 87.00 | 160.00 | 1.97 | 33.98 | 106.00 | 0.00 | 471.93 |
| 2.00 | 61.00 | 89.00 | 172.00 | 2.06 | 30.08 | 93.00 | 0.00 | 494.90 |
| 2.00 | 58.00 | 90.00 | 153.00 | 1.96 | 38.45 | 79.00 | 1.00 | 469.38 |
| 2.00 | 46.00 | 100.00 | 158.00 | 2.09 | 40.06 | 99.00 | 1.00 | 502.79 |

| | | | | | | | | |
|---|---|---|---|---|---|---|---|---|
| 2.00 | 57.00 | 84.00 | 162.00 | 1.94 | 32.01 | 96.00 | 1.00 | 466.61 |
| 2.00 | 44.00 | 80.00 | 160.00 | 1.89 | 31.25 | 79.00 | 1.00 | 452.55 |
| 2.00 | 60.00 | 71.00 | 172.00 | 1.84 | 24.00 | 85.00 | 1.00 | 442.03 |
| 2.00 | 37.00 | 69.00 | 149.00 | 1.69 | 31.08 | 81.00 | 0.00 | 405.58 |
| 2.00 | 42.00 | 62.00 | 155.00 | 1.63 | 25.81 | 96.00 | 1.00 | 392.12 |
| 2.00 | 49.00 | 84.00 | 152.00 | 1.88 | 36.36 | 79.00 | 1.00 | 451.98 |
| 2.00 | 42.00 | 70.00 | 163.00 | 1.78 | 26.35 | 76.00 | 1.00 | 427.27 |
| 2.00 | 55.00 | 68.00 | 167.00 | 1.78 | 24.38 | 75.00 | 1.00 | 426.26 |
| 2.00 | 42.00 | 77.00 | 160.00 | 1.85 | 30.08 | 99.00 | 1.00 | 443.98 |
| 2.00 | 47.00 | 58.00 | 165.00 | 1.63 | 21.30 | 88.00 | 1.00 | 391.31 |
| 2.00 | 40.00 | 60.00 | 164.00 | 1.65 | 22.31 | 94.00 | 1.00 | 396.79 |
| 2.00 | 39.00 | 89.00 | 172.00 | 2.06 | 30.08 | 90.00 | 1.00 | 494.90 |
| 2.00 | 62.00 | 75.00 | 158.00 | 1.81 | 30.04 | 85.00 | 1.00 | 435.43 |
| 2.00 | 43.00 | 79.00 | 155.00 | 1.84 | 32.88 | 100.00 | 0.00 | 442.63 |
| 2.00 | 58.00 | 84.00 | 160.00 | 1.93 | 32.81 | 88.00 | 1.00 | 463.72 |

| Cyclophosphamide | Paclitaxel | BNP_before | BNP_after | MDA_befc | MDA_afte | TNF_befor | TNF_after |
|---|---|---|---|---|---|---|---|
| 3783.97 | 1513.59 | 58.32 | 28.49 | 7.60 | 5.72 | 69.75 | 158.50 |
| 4682.91 | 1873.16 | 30.04 | 70.49 | 4.67 | 8.96 | 66.42 | 136.33 |
| 4859.30 | 1943.72 | 67.59 | 70.65 | 7.09 | 10.63 | 82.75 | 155.58 |
| 4003.40 | 1601.36 | 40.37 | 113.47 | 10.24 | 7.82 | 33.83 | 54.58 |
| 4406.54 | 1762.62 | 94.59 | 136.06 | 8.97 | 9.12 | 90.17 | 137.33 |
| 4174.21 | 1669.68 | 49.63 | 22.78 | 8.60 | 11.28 | 91.33 | 208.17 |
| 4383.24 | 1753.30 | 56.31 | 113.21 | 5.92 | 7.63 | 67.75 | 100.25 |
| 4593.56 | 1837.42 | 20.14 | 46.73 | 8.40 | 9.50 | 36.83 | 86.08 |
| 4452.95 | 1781.18 | 20.65 | 30.63 | 7.96 | 7.43 | 73.33 | 142.83 |
| 4724.40 | 1889.76 | 41.84 | 120.00 | 4.33 | 6.72 | 52.00 | 88.08 |
| 4352.47 | 1740.99 | 59.31 | 115.36 | 8.40 | 6.72 | 89.83 | 132.33 |
| 5075.43 | 2030.17 | 47.78 | 140.92 | 7.62 | 5.20 | 83.08 | 180.67 |
| 4448.28 | 1779.31 | 53.51 | 166.10 | 6.77 | 9.76 | 47.08 | 76.60 |
| 4591.30 | 1836.52 | 67.51 | 50.94 | 10.89 | 10.60 | 95.25 | 86.58 |
| 5245.95 | 2098.38 | 57.82 | 61.90 | 10.79 | 10.50 | 51.08 | 119.25 |
| 3740.59 | 1496.24 | 52.94 | 65.12 | 6.50 | 9.83 | 43.67 | 101.30 |
| 4198.29 | 1679.31 | 79.16 | 56.91 | 8.35 | 8.62 | 68.08 | 146.17 |
| 4945.79 | 1978.31 | 75.10 | 113.29 | 7.50 | 6.91 | 68.75 | 72.92 |
| 5127.49 | 2051.00 | 55.43 | 44.24 | 5.14 | 6.17 | 55.10 | 84.00 |
| 4863.58 | 1945.43 | 73.45 | 48.31 | 4.52 | 8.47 | 39.58 | 171.33 |
| 4635.52 | 1854.21 | 55.14 | 100.69 | 6.30 | 8.70 | 79.83 | 162.67 |
| 4047.72 | 1619.09 | 68.32 | 90.74 | 7.41 | 9.70 | 68.11 | 140.40 |
| 4061.33 | 1624.53 | 45.37 | 110.97 | 4.98 | 8.30 | 75.30 | 155.70 |
| 4692.12 | 1876.85 | 47.36 | 112.00 | 5.30 | 7.50 | 80.40 | 133.10 |
| 4276.63 | 1710.65 | 59.31 | 100.30 | 8.50 | 9.60 | 85.50 | 125.00 |
| 4791.99 | 1916.80 | 71.30 | 120.10 | 8.20 | 10.10 | 55.70 | 110.60 |
| 4878.03 | 1951.21 | 82.00 | 114.60 | 8.70 | 7.00 | 89.30 | 95.50 |
| 4623.94 | 1849.58 | 73.40 | 110.90 | 6.30 | 9.90 | 47.20 | 86.40 |
| 4454.75 | 1781.90 | 33.90 | 85.70 | 5.50 | 7.70 | 61.30 | 112.00 |
| 4836.53 | 1934.61 | 27.30 | 77.10 | 6.90 | 8.20 | 103.00 | 104.20 |
| 4395.45 | 1758.18 | 76.20 | 103.00 | 4.80 | 7.30 | 32.10 | 70.90 |
| 4859.63 | 1943.85 | 84.10 | 89.50 | 5.90 | 6.00 | 77.00 | 118.30 |
| 5372.52 | 2149.01 | 70.36 | 52.43 | 3.90 | 4.73 | 103.25 | 75.25 |
| 4776.61 | 1910.64 | 85.98 | 78.55 | 4.70 | 3.99 | 96.70 | 63.90 |
| 4960.48 | 1984.19 | 44.24 | 29.94 | 3.50 | 4.30 | 52.92 | 94.75 |
| 3939.95 | 1575.98 | 80.94 | 41.91 | 5.60 | 4.20 | 54.00 | 87.00 |
| 4127.95 | 1651.18 | 33.96 | 75.86 | 5.50 | 5.60 | 70.67 | 95.58 |
| 4381.78 | 1752.71 | 44.92 | 29.39 | 7.80 | 4.30 | 74.67 | 35.50 |
| 4552.98 | 1821.19 | 79.08 | 60.51 | 7.50 | 6.80 | 81.17 | 82.17 |
| 4084.70 | 1633.88 | 45.02 | 49.22 | 7.30 | 5.03 | 64.67 | 155.00 |
| 3764.25 | 1505.70 | 39.10 | 79.63 | 4.40 | 3.14 | 94.50 | 151.67 |
| 5267.26 | 2106.90 | 34.98 | 54.00 | 7.90 | 4.80 | 19.25 | 62.00 |
| 4812.65 | 1925.06 | 77.86 | 42.51 | 9.20 | 8.07 | 76.30 | 45.00 |
| 4478.04 | 1791.21 | 87.27 | 55.76 | 11.52 | 7.90 | 128.75 | 30.33 |
| 4338.85 | 1735.54 | 47.55 | 31.12 | 8.60 | 6.51 | 108.42 | 62.58 |
| 4719.32 | 1887.73 | 22.04 | 42.24 | 7.30 | 8.06 | 30.75 | 37.58 |
| 4949.02 | 1979.61 | 82.22 | 69.84 | 6.50 | 4.64 | 106.00 | 37.92 |
| 4693.83 | 1877.53 | 97.43 | 85.35 | 7.80 | 9.00 | 100.08 | 11.17 |
| 5027.92 | 2011.17 | 59.47 | 39.87 | 10.20 | 6.10 | 93.50 | 51.25 |

| | | | | | | | |
|---|---|---|---|---|---|---|---|
| 4666.13 | 1866.45 | 16.84 | 17.00 | 6.90 | 4.70 | 95.17 | 43.75 |
| 4525.48 | 1810.19 | 73.73 | 32.12 | 9.30 | 7.60 | 28.08 | 51.08 |
| 4420.32 | 1768.13 | 74.36 | 45.73 | 7.70 | 9.80 | 36.42 | 69.17 |
| 4055.81 | 1622.32 | 75.98 | 57.18 | 6.70 | 8.30 | 63.83 | 111.08 |
| 3921.22 | 1568.49 | 67.47 | 70.21 | 7.10 | 5.40 | 81.40 | 127.30 |
| 4519.82 | 1807.93 | 55.32 | 50.39 | 8.50 | 7.50 | 70.00 | 139.10 |
| 4272.70 | 1709.08 | 84.91 | 55.94 | 5.10 | 4.40 | 78.60 | 120.00 |
| 4262.58 | 1705.03 | 44.36 | 41.97 | 4.10 | 7.70 | 69.20 | 130.90 |
| 4439.82 | 1775.93 | 35.40 | 20.00 | 6.30 | 8.00 | 69.30 | 99.10 |
| 3913.06 | 1565.22 | 65.90 | 44.80 | 7.50 | 5.10 | 81.40 | 60.70 |
| 3967.87 | 1587.15 | 73.60 | 50.70 | 4.80 | 3.70 | 91.70 | 84.30 |
| 4949.02 | 1979.61 | 27.00 | 23.70 | 6.10 | 6.80 | 33.60 | 67.30 |
| 4354.31 | 1741.72 | 28.30 | 55.70 | 5.50 | 4.10 | 39.80 | 55.30 |
| 4426.29 | 1770.51 | 25.80 | 41.20 | 9.70 | 7.50 | 110.20 | 97.20 |
| 4637.24 | 1854.90 | 31.80 | 29.10 | 10.40 | 10.10 | 69.60 | 95.20 |

| NT_Before | NT_After | EF_before | EF_after | CTCEA_baseline | CTCEA_3 | CTCEA_6 | CTCEA_9 | CTCEA_12 |
|---|---|---|---|---|---|---|---|---|
| 95.20 | 127.00 | 66.00 | 60.00 | 0.00 | 1.00 | 1.00 | 3.00 | 2.00 |
| 57.70 | 93.30 | 70.00 | 66.00 | 0.00 | 1.00 | 1.00 | 2.00 | 1.00 |
| 41.80 | 55.20 | 65.00 | 61.00 | 0.00 | 2.00 | 3.00 | 3.00 | 3.00 |
| 46.30 | 72.20 | 67.00 | 61.00 | 0.00 | 1.00 | 1.00 | 1.00 | 2.00 |
| 47.20 | 71.60 | 63.00 | 60.00 | 0.00 | 2.00 | 2.00 | 3.00 | 3.00 |
| 75.40 | 70.70 | 66.00 | 62.00 | 0.00 | 1.00 | 1.00 | 1.00 | 2.00 |
| 57.20 | 97.00 | 68.00 | 62.00 | 0.00 | 1.00 | 1.00 | 2.00 | 2.00 |
| 53.10 | 138.30 | 61.00 | 57.00 | 0.00 | 2.00 | 2.00 | 2.00 | 2.00 |
| 49.60 | 65.50 | 69.00 | 61.00 | 0.00 | 1.00 | 1.00 | 2.00 | 1.00 |
| 59.70 | 106.50 | 61.00 | 59.00 | 0.00 | 1.00 | 1.00 | 3.00 | 2.00 |
| 71.70 | 44.20 | 66.00 | 59.00 | 0.00 | 2.00 | 2.00 | 2.00 | 2.00 |
| 64.10 | 89.30 | 68.00 | 62.00 | 0.00 | 1.00 | 2.00 | 2.00 | 2.00 |
| 51.70 | 82.10 | 73.00 | 66.00 | 0.00 | 1.00 | 1.00 | 1.00 | 1.00 |
| 66.30 | 81.10 | 62.00 | 66.00 | 0.00 | 2.00 | 2.00 | 2.00 | 2.00 |
| 97.70 | 76.50 | 69.00 | 58.00 | 0.00 | 1.00 | 2.00 | 1.00 | 2.00 |
| 87.30 | 87.90 | 66.00 | 60.00 | 0.00 | 2.00 | 1.00 | 2.00 | 2.00 |
| 69.60 | 95.40 | 67.00 | 64.00 | 0.00 | 2.00 | 2.00 | 2.00 | 3.00 |
| 97.30 | 105.00 | 69.00 | 61.00 | 0.00 | 1.00 | 1.00 | 1.00 | 2.00 |
| 94.80 | 77.40 | 66.00 | 59.00 | 0.00 | 2.00 | 2.00 | 3.00 | 3.00 |
| 53.40 | 107.80 | 67.00 | 63.00 | 0.00 | 2.00 | 1.00 | 2.00 | 2.00 |
| 61.80 | 93.40 | 69.00 | 65.00 | 0.00 | 1.00 | 2.00 | 3.00 | 3.00 |
| 48.30 | 92.00 | 67.00 | 60.00 | 0.00 | 1.00 | 1.00 | 2.00 | 2.00 |
| 65.40 | 103.80 | 68.00 | 61.00 | 0.00 | 1.00 | 2.00 | 3.00 | 3.00 |
| 58.50 | 94.60 | 68.00 | 55.00 | 0.00 | 1.00 | 2.00 | 3.00 | 2.00 |
| 80.80 | 99.20 | 67.00 | 62.00 | 0.00 | 1.00 | 1.00 | 1.00 | 1.00 |
| 80.90 | 61.20 | 60.00 | 55.00 | 0.00 | 1.00 | 1.00 | 1.00 | 1.00 |
| 86.50 | 105.40 | 66.00 | 67.00 | 0.00 | 1.00 | 1.00 | 1.00 | 2.00 |
| 64.50 | 95.80 | 70.00 | 68.00 | 0.00 | 2.00 | 2.00 | 2.00 | 3.00 |
| 79.30 | 88.60 | 67.00 | 65.00 | 0.00 | 1.00 | 1.00 | 2.00 | 2.00 |
| 80.90 | 93.90 | 67.00 | 62.00 | 0.00 | 2.00 | 2.00 | 2.00 | 3.00 |
| 65.30 | 70.20 | 70.00 | 63.00 | 0.00 | 1.00 | 1.00 | 1.00 | 2.00 |
| 75.20 | 92.10 | 69.00 | 64.00 | 0.00 | 1.00 | 1.00 | 1.00 | 1.00 |
| 91.50 | 75.80 | 59.00 | 55.00 | 0.00 | 2.00 | 2.00 | 3.00 | 3.00 |
| 84.30 | 68.10 | 64.00 | 57.00 | 0.00 | 1.00 | 1.00 | 1.00 | 1.00 |
| 59.10 | 61.60 | 70.00 | 68.00 | 0.00 | 1.00 | 2.00 | 2.00 | 2.00 |
| 42.10 | 69.10 | 66.00 | 56.00 | 0.00 | 1.00 | 1.00 | 2.00 | 2.00 |
| 34.00 | 62.40 | 69.00 | 67.00 | 0.00 | 2.00 | 2.00 | 2.00 | 2.00 |
| 51.90 | 66.00 | 61.00 | 58.00 | 0.00 | 1.00 | 1.00 | 2.00 | 2.00 |
| 44.70 | 65.50 | 66.00 | 69.00 | 0.00 | 1.00 | 1.00 | 1.00 | 2.00 |
| 60.40 | 46.00 | 58.00 | 56.00 | 0.00 | 1.00 | 1.00 | 2.00 | 2.00 |
| 69.40 | 63.10 | 64.00 | 60.00 | 0.00 | 1.00 | 1.00 | 1.00 | 1.00 |
| 61.40 | 51.20 | 70.00 | 66.00 | 0.00 | 1.00 | 1.00 | 2.00 | 2.00 |
| 98.40 | 66.20 | 64.00 | 57.00 | 0.00 | 1.00 | 1.00 | 1.00 | 1.00 |
| 102.90 | 77.40 | 64.00 | 59.00 | 0.00 | 1.00 | 1.00 | 2.00 | 2.00 |
| 49.90 | 41.30 | 70.00 | 68.00 | 0.00 | 2.00 | 2.00 | 1.00 | 2.00 |
| 73.40 | 54.00 | 66.00 | 58.00 | 0.00 | 1.00 | 1.00 | 1.00 | 1.00 |
| 78.50 | 66.40 | 69.00 | 65.00 | 0.00 | 2.00 | 2.00 | 2.00 | 2.00 |
| 68.90 | 44.40 | 59.00 | 55.00 | 0.00 | 1.00 | 1.00 | 2.00 | 2.00 |
| 88.00 | 53.70 | 66.00 | 64.00 | 0.00 | 1.00 | 1.00 | 1.00 | 1.00 |

| | | | | | | | | |
|---|---|---|---|---|---|---|---|---|
| 41.30 | 40.80 | 61.00 | 55.00 | 0.00 | 2.00 | 2.00 | 3.00 | 3.00 |
| 89.50 | 65.00 | 69.00 | 61.00 | 0.00 | 1.00 | 1.00 | 2.00 | 2.00 |
| 99.60 | 73.90 | 65.00 | 57.00 | 0.00 | 2.00 | 1.00 | 1.00 | 1.00 |
| 54.40 | 49.00 | 69.00 | 66.00 | 0.00 | 1.00 | 1.00 | 2.00 | 2.00 |
| 70.90 | 55.00 | 67.00 | 65.00 | 0.00 | 1.00 | 1.00 | 1.00 | 1.00 |
| 54.70 | 50.90 | 67.00 | 60.00 | 0.00 | 1.00 | 1.00 | 2.00 | 2.00 |
| 88.70 | 64.00 | 65.00 | 58.00 | 0.00 | 1.00 | 1.00 | 1.00 | 1.00 |
| 81.00 | 41.90 | 61.00 | 56.00 | 0.00 | 1.00 | 1.00 | 1.00 | 2.00 |
| 49.30 | 43.80 | 64.00 | 58.00 | 0.00 | 1.00 | 1.00 | 1.00 | 1.00 |
| 46.90 | 50.40 | 61.00 | 57.00 | 0.00 | 1.00 | 1.00 | 1.00 | 1.00 |
| 88.00 | 72.30 | 68.00 | 61.00 | 0.00 | 1.00 | 1.00 | 2.00 | 2.00 |
| 94.10 | 77.20 | 71.00 | 69.00 | 0.00 | 1.00 | 1.00 | 1.00 | 1.00 |
| 65.40 | 60.50 | 59.00 | 55.00 | 0.00 | 1.00 | 2.00 | 2.00 | 2.00 |
| 68.10 | 70.90 | 58.00 | 55.00 | 0.00 | 1.00 | 1.00 | 1.00 | 2.00 |
| 95.40 | 75.70 | 70.00 | 62.00 | 0.00 | 1.00 | 1.00 | 1.00 | 1.00 |

| NTX12_base | NTX12_1st | NTX12_2nd | NTX12_3rd | NTX12_4th | Menopaus_stat | Stage | Surgery |
|---|---|---|---|---|---|---|---|
| 45.00 | 37.00 | 34.00 | 28.00 | 26.00 | 0.00 | 2.00 | 1.00 |
| 46.00 | 37.00 | 34.00 | 30.00 | 26.00 | 0.00 | 1.00 | 1.00 |
| 46.00 | 32.00 | 30.00 | 31.00 | 26.00 | 0.00 | 2.00 | 1.00 |
| 45.00 | 35.00 | 31.00 | 31.00 | 28.00 | 0.00 | 1.00 | 1.00 |
| 42.00 | 31.00 | 29.00 | 32.00 | 29.00 | 1.00 | 2.00 | 2.00 |
| 44.00 | 31.00 | 30.00 | 30.00 | 27.00 | 1.00 | 1.00 | 2.00 |
| 45.00 | 35.00 | 33.00 | 32.00 | 30.00 | 1.00 | 1.00 | 2.00 |
| 48.00 | 35.00 | 34.00 | 29.00 | 29.00 | 0.00 | 1.00 | 1.00 |
| 46.00 | 31.00 | 26.00 | 33.00 | 29.00 | 0.00 | 2.00 | 1.00 |
| 44.00 | 39.00 | 36.00 | 33.00 | 27.00 | 1.00 | 2.00 | 2.00 |
| 43.00 | 35.00 | 33.00 | 34.00 | 30.00 | 1.00 | 1.00 | 2.00 |
| 47.00 | 38.00 | 37.00 | 34.00 | 30.00 | 0.00 | 1.00 | 1.00 |
| 43.00 | 38.00 | 36.00 | 34.00 | 27.00 | 0.00 | 1.00 | 1.00 |
| 46.00 | 34.00 | 33.00 | 30.00 | 26.00 | 0.00 | 2.00 | 1.00 |
| 46.00 | 36.00 | 32.00 | 30.00 | 26.00 | 0.00 | 2.00 | 1.00 |
| 44.00 | 34.00 | 32.00 | 30.00 | 27.00 | 1.00 | 1.00 | 2.00 |
| 44.00 | 36.00 | 34.00 | 30.00 | 28.00 | 1.00 | 1.00 | 2.00 |
| 47.00 | 38.00 | 35.00 | 31.00 | 27.00 | 0.00 | 2.00 | 1.00 |
| 45.00 | 33.00 | 29.00 | 27.00 | 26.00 | 0.00 | 1.00 | 1.00 |
| 44.00 | 40.00 | 36.00 | 27.00 | 26.00 | 1.00 | 2.00 | 2.00 |
| 46.00 | 32.00 | 30.00 | 32.00 | 29.00 | 0.00 | 1.00 | 1.00 |
| 47.00 | 35.00 | 33.00 | 34.00 | 31.00 | 1.00 | 2.00 | 2.00 |
| 46.00 | 35.00 | 30.00 | 30.00 | 27.00 | 0.00 | 1.00 | 1.00 |
| 43.00 | 34.00 | 32.00 | 32.00 | 30.00 | 1.00 | 2.00 | 2.00 |
| 47.00 | 40.00 | 38.00 | 29.00 | 29.00 | 0.00 | 2.00 | 1.00 |
| 45.00 | 35.00 | 35.00 | 30.00 | 26.00 | 0.00 | 1.00 | 1.00 |
| 45.00 | 37.00 | 32.00 | 29.00 | 27.00 | 0.00 | 1.00 | 1.00 |
| 43.00 | 35.00 | 32.00 | 27.00 | 27.00 | 1.00 | 2.00 | 2.00 |
| 47.00 | 40.00 | 29.00 | 28.00 | 27.00 | 1.00 | 2.00 | 2.00 |
| 43.00 | 33.00 | 29.00 | 27.00 | 25.00 | 1.00 | 2.00 | 2.00 |
| 45.00 | 40.00 | 31.00 | 29.00 | 29.00 | 0.00 | 1.00 | 1.00 |
| 45.00 | 37.00 | 36.00 | 26.00 | 24.00 | 1.00 | 1.00 | 2.00 |
| 47.00 | 35.00 | 31.00 | 30.00 | 26.00 | 0.00 | 2.00 | 1.00 |
| 43.00 | 37.00 | 32.00 | 33.00 | 32.00 | 0.00 | 2.00 | 1.00 |
| 46.00 | 40.00 | 33.00 | 28.00 | 25.00 | 0.00 | 2.00 | 1.00 |
| 46.00 | 32.00 | 32.00 | 30.00 | 26.00 | 0.00 | 2.00 | 1.00 |
| 43.00 | 40.00 | 36.00 | 27.00 | 28.00 | 1.00 | 1.00 | 2.00 |
| 47.00 | 37.00 | 33.00 | 28.00 | 27.00 | 1.00 | 2.00 | 2.00 |
| 44.00 | 41.00 | 34.00 | 33.00 | 29.00 | 0.00 | 1.00 | 1.00 |
| 46.00 | 37.00 | 36.00 | 33.00 | 29.00 | 0.00 | 1.00 | 1.00 |
| 47.00 | 37.00 | 33.00 | 26.00 | 28.00 | 0.00 | 2.00 | 1.00 |
| 47.00 | 34.00 | 34.00 | 32.00 | 29.00 | 0.00 | 2.00 | 1.00 |
| 44.00 | 39.00 | 36.00 | 31.00 | 28.00 | 0.00 | 2.00 | 1.00 |
| 44.00 | 39.00 | 34.00 | 36.00 | 35.00 | 0.00 | 1.00 | 1.00 |
| 46.00 | 33.00 | 34.00 | 35.00 | 32.00 | 1.00 | 1.00 | 2.00 |
| 46.00 | 35.00 | 32.00 | 35.00 | 31.00 | 1.00 | 1.00 | 2.00 |
| 45.00 | 39.00 | 35.00 | 33.00 | 31.00 | 1.00 | 1.00 | 2.00 |
| 48.00 | 37.00 | 31.00 | 30.00 | 29.00 | 0.00 | 1.00 | 1.00 |
| 46.00 | 39.00 | 35.00 | 33.00 | 32.00 | 0.00 | 2.00 | 1.00 |

| | | | | | | | |
|---|---|---|---|---|---|---|---|
| 44.00 | 33.00 | 35.00 | 34.00 | 32.00 | 1.00 | 2.00 | 2.00 |
| 43.00 | 38.00 | 29.00 | 28.00 | 27.00 | 1.00 | 2.00 | 2.00 |
| 48.00 | 38.00 | 29.00 | 35.00 | 32.00 | 1.00 | 2.00 | 2.00 |
| 45.00 | 36.00 | 31.00 | 27.00 | 26.00 | 0.00 | 2.00 | 1.00 |
| 45.00 | 36.00 | 35.00 | 34.00 | 28.00 | 0.00 | 1.00 | 1.00 |
| 45.00 | 38.00 | 37.00 | 29.00 | 28.00 | 0.00 | 1.00 | 1.00 |
| 44.00 | 36.00 | 36.00 | 33.00 | 31.00 | 1.00 | 1.00 | 2.00 |
| 45.00 | 32.00 | 33.00 | 36.00 | 33.00 | 0.00 | 2.00 | 1.00 |
| 44.00 | 38.00 | 32.00 | 35.00 | 31.00 | 0.00 | 1.00 | 1.00 |
| 45.00 | 36.00 | 30.00 | 31.00 | 30.00 | 1.00 | 1.00 | 2.00 |
| 44.00 | 33.00 | 29.00 | 32.00 | 30.00 | 0.00 | 1.00 | 1.00 |
| 47.00 | 37.00 | 31.00 | 29.00 | 26.00 | 1.00 | 2.00 | 2.00 |
| 43.00 | 31.00 | 29.00 | 32.00 | 27.00 | 0.00 | 2.00 | 1.00 |
| 42.00 | 33.00 | 30.00 | 32.00 | 28.00 | 1.00 | 1.00 | 2.00 |
| 46.00 | 30.00 | 29.00 | 34.00 | 30.00 | 0.00 | 1.00 | 1.00 |

| GIT_SE | Nausea | Abd_Pain | dizziness | Headache | Fatigue_G | Fatigue_G | Neutropenia | Leukopenia |
|---|---|---|---|---|---|---|---|---|
| 1.00 | 1.00 | 0.00 | 0.00 | 0.00 | 1.00 | 0.00 | 1.00 | 0.00 |
| 0.00 | 0.00 | 0.00 | 1.00 | 0.00 | 1.00 | 0.00 | 0.00 | 0.00 |
| 0.00 | 0.00 | 0.00 | 0.00 | 0.00 | 0.00 | 0.00 | 0.00 | 1.00 |
| 0.00 | 0.00 | 0.00 | 0.00 | 0.00 | 1.00 | 0.00 | 0.00 | 0.00 |
| 0.00 | 0.00 | 0.00 | 0.00 | 0.00 | 1.00 | 0.00 | 0.00 | 1.00 |
| 0.00 | 0.00 | 0.00 | 0.00 | 0.00 | 1.00 | 0.00 | 0.00 | 0.00 |
| 0.00 | 0.00 | 0.00 | 0.00 | 0.00 | 0.00 | 1.00 | 0.00 | 0.00 |
| 0.00 | 0.00 | 0.00 | 0.00 | 0.00 | 1.00 | 0.00 | 0.00 | 0.00 |
| 0.00 | 0.00 | 0.00 | 0.00 | 0.00 | 1.00 | 0.00 | 0.00 | 1.00 |
| 0.00 | 0.00 | 0.00 | 0.00 | 1.00 | 0.00 | 0.00 | 0.00 | 0.00 |
| 0.00 | 0.00 | 0.00 | 0.00 | 0.00 | 0.00 | 0.00 | 1.00 | 0.00 |
| 0.00 | 0.00 | 0.00 | 0.00 | 0.00 | 1.00 | 0.00 | 0.00 | 1.00 |
| 1.00 | 0.00 | 1.00 | 0.00 | 0.00 | 1.00 | 0.00 | 1.00 | 0.00 |
| 1.00 | 1.00 | 0.00 | 0.00 | 0.00 | 1.00 | 0.00 | 0.00 | 0.00 |
| 0.00 | 0.00 | 0.00 | 0.00 | 0.00 | 1.00 | 0.00 | 0.00 | 1.00 |
| 0.00 | 0.00 | 0.00 | 1.00 | 0.00 | 1.00 | 0.00 | 1.00 | 0.00 |
| 0.00 | 0.00 | 0.00 | 0.00 | 0.00 | 1.00 | 0.00 | 0.00 | 0.00 |
| 0.00 | 0.00 | 0.00 | 0.00 | 0.00 | 1.00 | 0.00 | 1.00 | 0.00 |
| 0.00 | 0.00 | 0.00 | 0.00 | 0.00 | 1.00 | 0.00 | 0.00 | 1.00 |
| 0.00 | 0.00 | 0.00 | 0.00 | 0.00 | 1.00 | 0.00 | 0.00 | 0.00 |
| 0.00 | 0.00 | 0.00 | 0.00 | 0.00 | 1.00 | 0.00 | 0.00 | 0.00 |
| 0.00 | 0.00 | 0.00 | 0.00 | 0.00 | 1.00 | 0.00 | 0.00 | 1.00 |
| 0.00 | 0.00 | 0.00 | 0.00 | 0.00 | 1.00 | 0.00 | 0.00 | 0.00 |
| 0.00 | 0.00 | 0.00 | 0.00 | 0.00 | 0.00 | 1.00 | 0.00 | 1.00 |
| 0.00 | 0.00 | 0.00 | 0.00 | 0.00 | 1.00 | 0.00 | 0.00 | 1.00 |
| 0.00 | 0.00 | 0.00 | 0.00 | 0.00 | 1.00 | 0.00 | 0.00 | 0.00 |
| 0.00 | 0.00 | 0.00 | 0.00 | 0.00 | 1.00 | 0.00 | 0.00 | 0.00 |
| 0.00 | 0.00 | 0.00 | 0.00 | 0.00 | 1.00 | 0.00 | 0.00 | 0.00 |
| 0.00 | 0.00 | 0.00 | 1.00 | 0.00 | 1.00 | 0.00 | 0.00 | 1.00 |
| 0.00 | 0.00 | 0.00 | 0.00 | 0.00 | 1.00 | 0.00 | 1.00 | 0.00 |
| 0.00 | 0.00 | 0.00 | 0.00 | 1.00 | 1.00 | 0.00 | 0.00 | 0.00 |
| 0.00 | 0.00 | 0.00 | 0.00 | 0.00 | 1.00 | 0.00 | 0.00 | 1.00 |
| 0.00 | 0.00 | 0.00 | 0.00 | 0.00 | 1.00 | 0.00 | 0.00 | 0.00 |
| 0.00 | 0.00 | 0.00 | 0.00 | 0.00 | 1.00 | 0.00 | 0.00 | 0.00 |
| 0.00 | 0.00 | 0.00 | 0.00 | 0.00 | 1.00 | 0.00 | 0.00 | 1.00 |
| 1.00 | 1.00 | 0.00 | 0.00 | 0.00 | 1.00 | 0.00 | 1.00 | 0.00 |
| 0.00 | 0.00 | 0.00 | 0.00 | 0.00 | 1.00 | 0.00 | 0.00 | 0.00 |
| 0.00 | 0.00 | 0.00 | 0.00 | 0.00 | 1.00 | 0.00 | 0.00 | 1.00 |
| 0.00 | 0.00 | 0.00 | 0.00 | 0.00 | 0.00 | 0.00 | 0.00 | 1.00 |
| 1.00 | 0.00 | 1.00 | 0.00 | 0.00 | 1.00 | 0.00 | 1.00 | 0.00 |
| 0.00 | 0.00 | 0.00 | 0.00 | 0.00 | 1.00 | 0.00 | 1.00 | 0.00 |
| 0.00 | 0.00 | 0.00 | 0.00 | 0.00 | 1.00 | 0.00 | 0.00 | 0.00 |
| 0.00 | 0.00 | 0.00 | 0.00 | 0.00 | 1.00 | 0.00 | 0.00 | 1.00 |
| 0.00 | 0.00 | 0.00 | 0.00 | 0.00 | 1.00 | 0.00 | 0.00 | 0.00 |
| 0.00 | 0.00 | 0.00 | 0.00 | 0.00 | 1.00 | 0.00 | 0.00 | 0.00 |
| 0.00 | 0.00 | 0.00 | 0.00 | 0.00 | 0.00 | 0.00 | 0.00 | 1.00 |
| 1.00 | 0.00 | 0.00 | 0.00 | 0.00 | 0.00 | 0.00 | 0.00 | 1.00 |
| 0.00 | 0.00 | 1.00 | 0.00 | 0.00 | 0.00 | 0.00 | 0.00 | 0.00 |
| 0.00 | 0.00 | 0.00 | 0.00 | 0.00 | 0.00 | 0.00 | 1.00 | 0.00 |

| | | | | | | | | |
|---|---|---|---|---|---|---|---|---|
| 0.00 | 0.00 | 0.00 | 0.00 | 1.00 | 1.00 | 0.00 | 0.00 | 0.00 |
| 0.00 | 0.00 | 0.00 | 0.00 | 0.00 | 1.00 | 0.00 | 1.00 | 0.00 |
| 1.00 | 1.00 | 0.00 | 0.00 | 0.00 | 1.00 | 0.00 | 0.00 | 1.00 |
| 0.00 | 0.00 | 0.00 | 0.00 | 0.00 | 1.00 | 0.00 | 0.00 | 0.00 |
| 0.00 | 0.00 | 0.00 | 0.00 | 0.00 | 0.00 | 0.00 | 1.00 | 0.00 |
| 0.00 | 0.00 | 0.00 | 1.00 | 0.00 | 1.00 | 0.00 | 0.00 | 0.00 |
| 0.00 | 0.00 | 0.00 | 0.00 | 0.00 | 1.00 | 0.00 | 0.00 | 0.00 |
| 0.00 | 0.00 | 0.00 | 0.00 | 1.00 | 1.00 | 0.00 | 1.00 | 0.00 |
| 0.00 | 0.00 | 0.00 | 0.00 | 0.00 | 0.00 | 0.00 | 0.00 | 1.00 |
| 0.00 | 0.00 | 0.00 | 0.00 | 1.00 | 1.00 | 0.00 | 0.00 | 0.00 |
| 0.00 | 0.00 | 0.00 | 0.00 | 0.00 | 1.00 | 0.00 | 0.00 | 0.00 |
| 0.00 | 0.00 | 0.00 | 0.00 | 0.00 | 1.00 | 0.00 | 0.00 | 1.00 |
| 0.00 | 0.00 | 0.00 | 0.00 | 0.00 | 1.00 | 0.00 | 0.00 | 0.00 |
| 0.00 | 0.00 | 0.00 | 1.00 | 0.00 | 0.00 | 0.00 | 0.00 | 0.00 |
| 0.00 | 0.00 | 0.00 | 0.00 | 0.00 | 1.00 | 0.00 | 0.00 | 0.00 |

| Infection | Grade_1 | Grade_2 | Grade_3 | Grad_1 | Grad_2 | Grad_3 | Gra_1 | Gra_2 | Gra_3 |
|---|---|---|---|---|---|---|---|---|---|
| 1.00 | 0.00 | 0.00 | 1.00 | 1.00 | 0.00 | 0.00 | 1.00 | 0.00 | 0.00 |
| 0.00 | 0.00 | 0.00 | 1.00 | 1.00 | 0.00 | 0.00 | 1.00 | 0.00 | 0.00 |
| 0.00 | 0.00 | 0.00 | 1.00 | 1.00 | 0.00 | 0.00 | 1.00 | 0.00 | 0.00 |
| 0.00 | 0.00 | 0.00 | 1.00 | 1.00 | 0.00 | 0.00 | 1.00 | 0.00 | 0.00 |
| 0.00 | 0.00 | 0.00 | 1.00 | 1.00 | 0.00 | 0.00 | 1.00 | 0.00 | 0.00 |
| 0.00 | 0.00 | 0.00 | 1.00 | 1.00 | 0.00 | 0.00 | 1.00 | 0.00 | 0.00 |
| 1.00 | 0.00 | 0.00 | 1.00 | 1.00 | 0.00 | 0.00 | 1.00 | 0.00 | 0.00 |
| 0.00 | 0.00 | 0.00 | 1.00 | 1.00 | 0.00 | 0.00 | 1.00 | 0.00 | 0.00 |
| 1.00 | 1.00 | 0.00 | 0.00 | 1.00 | 0.00 | 0.00 | 1.00 | 0.00 | 0.00 |
| 0.00 | 1.00 | 0.00 | 0.00 | 1.00 | 0.00 | 0.00 | 1.00 | 0.00 | 0.00 |
| 0.00 | 1.00 | 0.00 | 0.00 | 0.00 | 1.00 | 0.00 | 1.00 | 0.00 | 0.00 |
| 1.00 | 1.00 | 0.00 | 0.00 | 0.00 | 1.00 | 0.00 | 1.00 | 0.00 | 0.00 |
| 0.00 | 1.00 | 0.00 | 0.00 | 0.00 | 1.00 | 0.00 | 1.00 | 0.00 | 0.00 |
| 0.00 | 1.00 | 0.00 | 0.00 | 0.00 | 1.00 | 0.00 | 1.00 | 0.00 | 0.00 |
| 0.00 | 0.00 | 1.00 | 0.00 | 0.00 | 1.00 | 0.00 | 1.00 | 0.00 | 0.00 |
| 0.00 | 0.00 | 1.00 | 0.00 | 0.00 | 1.00 | 0.00 | 1.00 | 0.00 | 0.00 |
| 0.00 | 0.00 | 1.00 | 0.00 | 0.00 | 1.00 | 0.00 | 1.00 | 0.00 | 0.00 |
| 0.00 | 0.00 | 1.00 | 0.00 | 0.00 | 1.00 | 0.00 | 1.00 | 0.00 | 0.00 |
| 1.00 | 0.00 | 1.00 | 0.00 | 0.00 | 1.00 | 0.00 | 0.00 | 1.00 | 0.00 |
| 0.00 | 0.00 | 1.00 | 0.00 | 0.00 | 1.00 | 0.00 | 0.00 | 1.00 | 0.00 |
| 0.00 | 0.00 | 1.00 | 0.00 | 0.00 | 1.00 | 0.00 | 0.00 | 1.00 | 0.00 |
| 0.00 | 0.00 | 1.00 | 0.00 | 0.00 | 1.00 | 0.00 | 0.00 | 1.00 | 0.00 |
| 0.00 | 0.00 | 1.00 | 0.00 | 0.00 | 1.00 | 0.00 | 0.00 | 1.00 | 0.00 |
| 0.00 | 0.00 | 1.00 | 0.00 | 0.00 | 1.00 | 0.00 | 0.00 | 1.00 | 0.00 |
| 0.00 | 0.00 | 1.00 | 0.00 | 0.00 | 0.00 | 1.00 | 0.00 | 1.00 | 0.00 |
| 0.00 | 0.00 | 1.00 | 0.00 | 0.00 | 0.00 | 1.00 | 0.00 | 1.00 | 0.00 |
| 0.00 | 0.00 | 1.00 | 0.00 | 0.00 | 0.00 | 1.00 | 0.00 | 1.00 | 0.00 |
| 0.00 | 0.00 | 1.00 | 0.00 | 0.00 | 0.00 | 1.00 | 0.00 | 1.00 | 0.00 |
| 0.00 | 0.00 | 1.00 | 0.00 | 0.00 | 0.00 | 1.00 | 0.00 | 1.00 | 0.00 |
| 0.00 | 0.00 | 1.00 | 0.00 | 0.00 | 0.00 | 1.00 | 0.00 | 1.00 | 0.00 |
| 0.00 | 0.00 | 1.00 | 0.00 | 0.00 | 0.00 | 1.00 | 0.00 | 1.00 | 0.00 |
| 0.00 | 0.00 | 1.00 | 0.00 | 0.00 | 0.00 | 1.00 | 0.00 | 0.00 | 1.00 |
| 0.00 | 0.00 | 0.00 | 1.00 | 1.00 | 0.00 | 0.00 | 1.00 | 0.00 | 0.00 |
| 0.00 | 0.00 | 0.00 | 1.00 | 1.00 | 0.00 | 0.00 | 1.00 | 0.00 | 0.00 |
| 1.00 | 0.00 | 1.00 | 0.00 | 1.00 | 0.00 | 0.00 | 1.00 | 0.00 | 0.00 |
| 0.00 | 0.00 | 1.00 | 0.00 | 1.00 | 0.00 | 0.00 | 1.00 | 0.00 | 0.00 |
| 0.00 | 0.00 | 1.00 | 0.00 | 1.00 | 0.00 | 0.00 | 1.00 | 0.00 | 0.00 |
| 1.00 | 0.00 | 1.00 | 0.00 | 1.00 | 0.00 | 0.00 | 1.00 | 0.00 | 0.00 |
| 0.00 | 0.00 | 1.00 | 0.00 | 1.00 | 0.00 | 0.00 | 1.00 | 0.00 | 0.00 |
| 0.00 | 0.00 | 1.00 | 0.00 | 1.00 | 0.00 | 0.00 | 1.00 | 0.00 | 0.00 |
| 0.00 | 0.00 | 1.00 | 0.00 | 1.00 | 0.00 | 0.00 | 1.00 | 0.00 | 0.00 |
| 0.00 | 0.00 | 1.00 | 0.00 | 1.00 | 0.00 | 0.00 | 1.00 | 0.00 | 0.00 |
| 1.00 | 0.00 | 1.00 | 0.00 | 1.00 | 0.00 | 0.00 | 1.00 | 0.00 | 0.00 |
| 0.00 | 0.00 | 1.00 | 0.00 | 1.00 | 0.00 | 0.00 | 1.00 | 0.00 | 0.00 |
| 0.00 | 0.00 | 1.00 | 0.00 | 1.00 | 0.00 | 0.00 | 1.00 | 0.00 | 0.00 |
| 0.00 | 0.00 | 1.00 | 0.00 | 1.00 | 0.00 | 0.00 | 1.00 | 0.00 | 0.00 |
| 0.00 | 0.00 | 1.00 | 0.00 | 1.00 | 0.00 | 0.00 | 1.00 | 0.00 | 0.00 |
| 0.00 | 0.00 | 1.00 | 0.00 | 1.00 | 0.00 | 0.00 | 1.00 | 0.00 | 0.00 |
| 0.00 | 0.00 | 1.00 | 0.00 | 0.00 | 1.00 | 0.00 | 1.00 | 0.00 | 0.00 |

| | | | | | | | | | |
|---|---|---|---|---|---|---|---|---|---|
| 0.00 | 0.00 | 1.00 | 0.00 | 0.00 | 1.00 | 0.00 | 1.00 | 0.00 | 0.00 |
| 0.00 | 0.00 | 1.00 | 0.00 | 0.00 | 1.00 | 0.00 | 1.00 | 0.00 | 0.00 |
| 0.00 | 0.00 | 1.00 | 0.00 | 0.00 | 1.00 | 0.00 | 1.00 | 0.00 | 0.00 |
| 0.00 | 1.00 | 0.00 | 0.00 | 0.00 | 1.00 | 0.00 | 1.00 | 0.00 | 0.00 |
| 0.00 | 1.00 | 0.00 | 0.00 | 0.00 | 1.00 | 0.00 | 1.00 | 0.00 | 0.00 |
| 0.00 | 1.00 | 0.00 | 0.00 | 0.00 | 1.00 | 0.00 | 1.00 | 0.00 | 0.00 |
| 0.00 | 1.00 | 0.00 | 0.00 | 0.00 | 1.00 | 0.00 | 1.00 | 0.00 | 0.00 |
| 0.00 | 1.00 | 0.00 | 0.00 | 0.00 | 1.00 | 0.00 | 1.00 | 0.00 | 0.00 |
| 0.00 | 1.00 | 0.00 | 0.00 | 0.00 | 1.00 | 0.00 | 0.00 | 1.00 | 0.00 |
| 0.00 | 1.00 | 0.00 | 0.00 | 0.00 | 1.00 | 0.00 | 0.00 | 1.00 | 0.00 |
| 0.00 | 1.00 | 0.00 | 0.00 | 0.00 | 1.00 | 0.00 | 0.00 | 1.00 | 0.00 |
| 0.00 | 1.00 | 0.00 | 0.00 | 0.00 | 1.00 | 0.00 | 0.00 | 1.00 | 0.00 |
| 0.00 | 1.00 | 0.00 | 0.00 | 0.00 | 1.00 | 0.00 | 0.00 | 1.00 | 0.00 |
| 0.00 | 1.00 | 0.00 | 0.00 | 0.00 | 0.00 | 1.00 | 0.00 | 1.00 | 0.00 |
| 0.00 | 1.00 | 0.00 | 0.00 | 0.00 | 0.00 | 1.00 | 0.00 | 1.00 | 0.00 |

| G_1 | G_2 | G_0 |
|---|---|---|
| 1.00 | 0.00 | 1.00 |
| 1.00 | 0.00 | 1.00 |
| 1.00 | 0.00 | 1.00 |
| 1.00 | 0.00 | 1.00 |
| 1.00 | 0.00 | 1.00 |
| 1.00 | 0.00 | 1.00 |
| 1.00 | 0.00 | 1.00 |
| 1.00 | 0.00 | 1.00 |
| 1.00 | 0.00 | 1.00 |
| 1.00 | 0.00 | 1.00 |
| 1.00 | 0.00 | 1.00 |
| 1.00 | 0.00 | 1.00 |
| 1.00 | 0.00 | 1.00 |
| 1.00 | 0.00 | 1.00 |
| 1.00 | 0.00 | 1.00 |
| 1.00 | 0.00 | 1.00 |
| 1.00 | 0.00 | 1.00 |
| 1.00 | 0.00 | 1.00 |
| 1.00 | 0.00 | 1.00 |
| 1.00 | 0.00 | 1.00 |
| 1.00 | 0.00 | 1.00 |
| 0.00 | 1.00 | 1.00 |
| 0.00 | 1.00 | 1.00 |
| 0.00 | 1.00 | 1.00 |
| 0.00 | 1.00 | 1.00 |
| 0.00 | 1.00 | 1.00 |
| 0.00 | 1.00 | 1.00 |
| 0.00 | 1.00 | 1.00 |
| 0.00 | 1.00 | 1.00 |
| 0.00 | 1.00 | 1.00 |
| 0.00 | 1.00 | 1.00 |
| 0.00 | 1.00 | 1.00 |
| 1.00 | 0.00 | 1.00 |
| 1.00 | 0.00 | 1.00 |
| 1.00 | 0.00 | 1.00 |
| 1.00 | 0.00 | 1.00 |
| 1.00 | 0.00 | 1.00 |
| 1.00 | 0.00 | 1.00 |
| 1.00 | 0.00 | 1.00 |
| 1.00 | 0.00 | 1.00 |
| 1.00 | 0.00 | 1.00 |
| 1.00 | 0.00 | 1.00 |
| 1.00 | 0.00 | 1.00 |
| 1.00 | 0.00 | 1.00 |
| 1.00 | 0.00 | 1.00 |
| 1.00 | 0.00 | 1.00 |
| 1.00 | 0.00 | 1.00 |
| 1.00 | 0.00 | 1.00 |
| 1.00 | 0.00 | 1.00 |

| | | |
|---|---|---|
| 1.00 | 0.00 | 1.00 |
| 1.00 | 0.00 | 1.00 |
| 1.00 | 0.00 | 1.00 |
| 1.00 | 0.00 | 1.00 |
| 1.00 | 0.00 | 1.00 |
| 1.00 | 0.00 | 1.00 |
| 1.00 | 0.00 | 1.00 |
| 1.00 | 0.00 | 1.00 |
| 1.00 | 0.00 | 1.00 |
| 0.00 | 1.00 | 1.00 |
| 0.00 | 1.00 | 1.00 |
| 0.00 | 1.00 | 1.00 |
| 0.00 | 1.00 | 1.00 |
| 0.00 | 1.00 | 1.00 |
| 0.00 | 1.00 | 1.00 |
